# Características e Tendências na Mortalidade em Diferentes Fenótipos de Insuficiência Cardíaca na Atenção Primária

**DOI:** 10.36660/abc.20190912

**Published:** 2021-08-09

**Authors:** Antonio José Lagoeiro Jorge, Leticia Mara dos Santos Barbetta, Eduardo Thadeu de Oliveira Correia, Wolney de Andrade Martins, Adson Renato Leite, Maria Auxiliadora Nogueira Saad, Márcia Maria Sales dos Santos, Dayse Mary Correia, Maria Luiza Garcia Rosa, Sérgio Chermont, Cárita Cunha dos Santos, Evandro Tinoco Mesquita

**Affiliations:** 1 Universidade Federal Fluminense NiteróiRJ Brasil Universidade Federal Fluminense, Niterói, RJ - Brasil.; 2 UHG C.T.E.B Brasil C.T.E.B, UHG, - Brasil.

**Keywords:** Insuficiência Cardíaca/tendências, Insuficiência Cardíaca/mortalidade, Prevalência, Atenção Primária a Saúde, Prognóstico, Epidemiologia, Volume Sistólico

## Abstract

**Fundamento:**

A classificação da insuficiência cardíaca (IC) por fenótipos possui grande relevância na prática clínica.

**Objetivo:**

O estudo visou analisar a prevalência, as características clínicas e os desfechos entre os fenótipos de IC no contexto da atenção primária.

**Métodos:**

Trata-se de uma análise de um estudo de coorte que incluiu 560 indivíduos, com idade ≥ 45 anos, que foram selecionados aleatoriamente em um programa de atenção primária. Todos os participantes foram submetidos a avaliações clínicas, dosagem do peptídeo natriurético tipo B (BNP), eletrocardiograma e ecocardiografia em um único dia. A IC com fração de ejeção do ventrículo esquerdo (FEVE) < 40% foi classificado como IC com fração de ejeção reduzida (ICFEr), FEVE de 40% a 49% como IC com fração de ejeção intermediária (ICFEi) e FEVE ≥ 50% como IC com fração de ejeção preservada (ICFEp). Após 5 anos, os pacientes foram reavaliados quanto à ocorrência do desfecho composto de óbito por qualquer causa ou internação por doença cardiovascular.

**Resultados:**

Dos 560 pacientes incluídos, 51 pacientes tinham IC (9,1%), 11 dos quais tinham ICFEr (21,6%), 10 tinham ICFEi (19,6%) e 30 tinham ICFEp (58,8%). A ICFEi foi semelhante à ICFEp nos níveis de BNP (p < 0,001), índice de massa do ventrículo esquerdo (p = 0,037) e índice de volume do átrio esquerdo (p < 0,001). O fenótipo de ICFEi foi semelhante ao de ICFEr em relação à doença arterial coronariana (p = 0,009). Após 5 anos, os pacientes com ICFEi apresentaram melhor prognóstico quando comparados aos pacientes com ICFEp e ICFEr (p < 0,001).

**Conclusão:**

A prevalência de ICFEI foi semelhante ao observado em estudos anteriores. A ICFEI apresentou características semelhantes a ICFEP neste estudo. Nossos dados mostram que a ICFEi teve melhor prognóstico em comparação com os outros dois fenótipos.

## Introdução

A classificação da insuficiência cardíaca (IC) por fenótipos possui grande relevância na prática clínica, uma vez que diferem em relação às características, ao prognóstico e ao tratamento do paciente.^1^ Classicamente, dois fenótipos de IC foram descritos nas diretrizes, a saber, IC com fração de ejeção reduzida (ICFEr) onde a fração de ejeção do ventrículo esquerdo (FEVE) é inferior a 50% e IC com fração de ejeção preservada (ICFEp) com FEVE ≥ 50%.^2^ Em 2013, a American College of Cardiology Foundation/American Heart Association publicou novas diretrizes para IC, nas quais os pacientes com FEVE entre 41% e 50% foram classificados como casos limítrofes de ICFEp.^3^ Em 2016, as diretrizes de IC da Sociedade Europeia de Cardiologia reconheceram a IC com FEVE entre 40% e 49% como um fenótipo distinto, denominado IC com fração de ejeção intermediária (ICFEi).^4^ Finalmente, em 2018, a Sociedade Brasileira de Cardiologia incluiu a ICFEi nas Diretrizes de Insuficiência Cardíaca Crônica e Aguda de 2018.^5^

Estudos recentes observaram que a prevalência de pacientes com ICFEi variou de 13% a 24% de todos os pacientes com IC.^6^^-^^8^ Dados atuais de estudos de IC indicam que a ICFEi apresenta características intermediárias.^8^ Além disso, uma metanálise que incluiu mais de 600.000 pacientes com IC concluiu que os pacientes com ICFEi apresentaram mortalidade por todas as causas mais baixa do que os pacientes com ICFEr e nenhuma diferença estatística dos pacientes com ICFEp. Em relação às internações por todas as causas, não houve diferença estatística entre os três fenótipos de IC.^9^

Não há estudos no Brasil que avaliem esse fenótipo na atenção primária. Portanto, o presente estudo visou analisar a prevalência e as características clínicas da ICFEi, bem como os desfechos entre os fenótipos de IC em pacientes no contexto da atenção primária.

## Métodos

Este estudo de coorte incluiu, na linha de base, 633 indivíduos com idade ≥ 45 anos, cadastrados no Programa de Atenção Primária do município de Niterói, município de médio porte com 487.562 habitantes no estado do Rio de Janeiro, Brasil. O Programa de Atenção Básica oferece cobertura a 137.463 residentes em 32 módulos de atendimento, divididos em 110 setores. Inicialmente, foram selecionados 21 setores por meio de uma sequência aleatória, gerada por um programa de computador, em que o peso de cada setor era proporcional ao número de indivíduos.^10^ Os dados foram coletados de julho de 2011 a dezembro de 2012. Após 5 anos, os pacientes neste estudo foram reavaliados quanto à ocorrência do desfecho composto de óbito por qualquer causa ou internação por doença cardiovascular. Durante o seguimento, ocorreram 73 (11,5%) perdas e o número final de indivíduos avaliados foi 560.

### População

**Figura 1:**
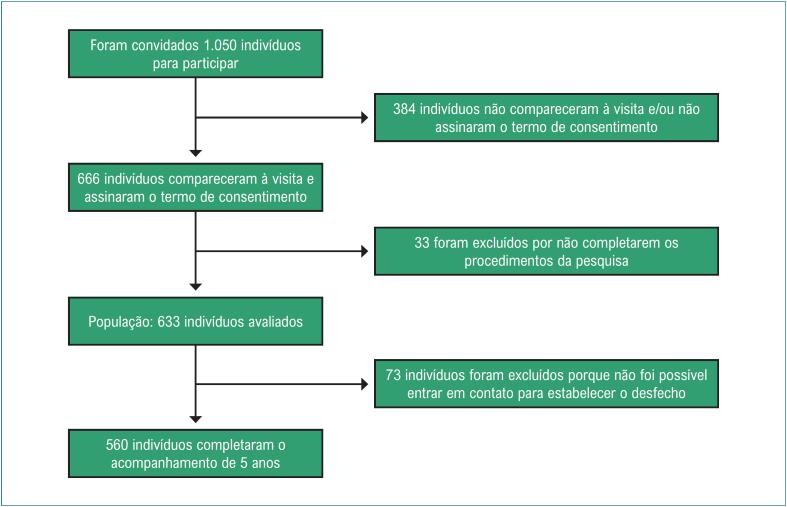
Fluxograma de seleção da população

O tamanho da amostra foi estimado a partir de uma prevalência mínima de IC de 6%, com um erro absoluto de 2% (intervalo de confiança = 99%, 4% a 8%). Tal suposição requeria um tamanho de amostra de 580 indivíduos. Em cada um dos 21 setores incluídos, foram aleatoriamente selecionados 30 indivíduos entre 45 e 100 anos de idade. Foram escolhidos 20 indivíduos adicionais por unidade, para permitir a substituição em caso de impossibilidade de participação, totalizando 1.050 indivíduos selecionados. Dessa forma, enviamos cartas aos funcionários das unidades de saúde para convidar 1.050 indivíduos a participarem deste estudo, sendo que 666 desses indivíduos compareceram à consulta e assinaram o termo de consentimento livre e esclarecido. Foram excluídos 33 indivíduos que não completaram todos os procedimentos da pesquisa. A população de base era de 633 indivíduos, no entanto, 73 (11,5%) não foram localizados após 5 anos e foram subsequentemente excluídos. A população final foi de 560 indivíduos. (Figura 1)

A escolha das unidades básicas de saúde e o número de indivíduos em cada unidade foram planejados de forma a representar a distribuição demográfica. A seleção dos sujeitos foi realizada por meio de uma sequência aleatória gerada por um programa de computador. Os critérios de inclusão foram idade ≥ 45 anos e disponibilidade em dar consentimento informado. Sempre que ocorria uma recusa, o próximo sujeito da lista aleatória era convidado a participar.

Todos os participantes do estudo foram submetidos a uma avaliação de um único dia que consistiu em: (a) anamnese e exames clínicos; (b) testes laboratoriais, incluindo dosagem do peptídeo natriurético do tipo B (BNP); (c) eletrocardiograma (ECG) de 12 derivações; e (d) ecocardiografia Doppler tecidual. Foi realizado o ECG em 12 derivações simultâneas. A ecocardiografia com Doppler tecidual foi realizada por dois médicos certificados, utilizando dois aparelhos portáteis, o Acuson Cypress 20 (Siemens, EUA) e o AU-3 Partner (Esaote, Itália). Os médicos foram cegados ao estado clínico e aos resultados dos exames. Os exames foram realizados de acordo com as recomendações de quantificação de câmaras da Sociedade Americana de Ecocardiografia e da Associação Europeia de Ecocardiografia.^11^ A função sistólica foi avaliada medindo a FEVE usando o método de Simpson.

### Definição dos fenótipos de insuficiência cardíaca

Foi confirmado o diagnóstico de ICFEr em indivíduos com histórico de IC ou a presença de sinais ou sintomas de IC e FEVE < 40%. Foi confirmado o diagnóstico de ICFEp em indivíduos com histórico de IC ou sinais ou sintomas de IC com FEVE ≥ 50% e índice de volume diastólico final (IVDF) < 97 mL/m^2^, na presença de disfunção diastólica do ventrículo esquerdo e BNP > 35 pg/mL. A ICFEi foi confirmada em indivíduos com histórico de IC ou sinais ou sintomas de IC com FEVE entre 40% e 49% e BNP > 35 pg/mL.^4^^,^^12^

### Análise estatística

As variáveis contínuas foram expressas como mediana e intervalo interquartil, uma vez que nenhuma delas foi positiva para normalidade quando testada pelo teste de Kolmogorov-Smirnov. As variáveis categóricas foram resumidas como frequências absolutas e relativas. Em relação às variáveis quantitativas, as diferenças entre os fenótipos de IC foram testadas com os testes não paramétricos (Mann-Whitney e Kruskal-Wallis), enquanto as variáveis categóricas foram avaliadas pelo teste de qui-quadrado. Foi estimada uma curva de Kaplan-Meier para os desfechos compostos das quatro possibilidades (sem IC, ICFEr, ICFEi e ICFEp). A diferença entre as quatro curvas e entre o grupo ICFEi e o grupo sem IC foi testada com o teste de log rank. Foram considerados estatisticamente significativos valores de p < 0,05. Todas as análises estatísticas foram realizadas com o software SPSS versão 23.0 (Chicago, Illinois, EUA).

### Considerações éticas

O presente estudo foi realizado de acordo com os princípios da Declaração de Helsinque revisada em 2000. O protocolo do estudo foi aprovado pelo Comitê de Ética institucional sob o número 0077.0.258.000-10.

## Resultados

### Prevalência e características de pacientes com ICFEi

Dos 560 pacientes incluídos no estudo, 509 não tinham o diagnóstico de IC (90,9%) e 51 foram diagnosticados com IC (9,1%). Dos 51 pacientes com IC, 11 tinham ICFEr (21,6%), 10 tinham ICFEi (19,6%) e 30 tinham ICFEp (58,8%). As características demográficas e clínicas dos pacientes com IC são apresentadas na Tabela 1. A ICFEi foi semelhante à ICFEp em relação ao índice de massa do ventrículo esquerdo (IMVE) e ao índice de volume do átrio esquerdo (IVAE). Observamos mais doença arterial coronariana em pacientes com fenótipo ICFEr, em comparação com ICFEi. O percentual de doença renal crônica foi intermediário no grupo ICFEi, sendo inferior ao grupo ICFEp e superior ao grupo ICFEr. O grupo ICFEi apresentou valores intermediários nas seguintes características: frequência cardíaca, glicemia e relação creatinina-albumina. Porém, não houve diferença estatística entre os grupos com IC com relação a essas características.

**Tabela 1:** Características demográficas e clínicas de pacientes com insuficiência cardíaca, de acordo com fenótipo ICFEp, ICFEi ou ICFEp

	Sem IC (n=509)	ICFEr (n=11)	ICFEi (n=10)	ICFEp (n=30)	Geral	ICFEr vs. ICFEi	ICFEp vs. ICFEi	ICFEr vs. ICFEp
Sexo masculino (%)	37	64	40	27	0,190	0,279	0,426	0,029
Idade, anos (mediana)	57(51-64)	74(57-78)	72(60-79)	72,5(64,7-81,7)	<0,001	0,809	0,708	0,871
IMC (mediana)	27,2(24,5-30,8)	24,9(21,3-25,9)	28,1(26,3-30,6)	26,9(22,0-30,7)	0,156	0,057	0,319	0,496
FC, bpm (mediana)	70,5(63,2-77,5)	69(55,5-72,5)	72 (62,1-79,1)	76,5(63,2-84,7)	0,360	0,324	0,573	0,108
PA sistólica, mmHg (mediana)	133,3(121-147,5)	146(116-161)	130(117,9-157,8)	151,7(135,2-179,7)	0,001	0,751	0,032	0,168
PA diastólica, mmHg (mediana)	82(74,1-90)	80(68,3-88,5)	77,5(71,1-90,9)	83,7(72,7-91,3)	0,699	0,778	0,699	0,310
BNP, pg/mL (mediana)	15(10-25)	306(153-615)	61,5(51-95)	87,5(52,7-120,5)	<0,0001	0,002	0,281	0,001
Glicose, mg/dL (mediana)	100(91-113)	103(84-119)	97(87-106,2)	100(94,7-119)	0,765	0,621	0,288	0,757
Ácido úrico, mg/dL (mediana)	5,1(4,2-6,1)	6,3(4,6-8,0)	5,2(4,9-6,5)	5,1(4,1-6,7)	0,192	0,398	0,430	0,108
Colesterol total, mg/dL (mediana)	213(186-244)	185(177-253)	199(180-240)	208(196-231)	0,629	0,623	0,453	0,502
Triglicerídeos, mg/dL (mediana)	118(86-169)	115(86-190)	106(66-152)	101(90-136)	0,481	0,571	0,851	0,482
Hemoglobina, g/dL (mediana)	13,7(12,8-14,7)	13,9(13,4-16,4)	13,7(12,1-14,3)	13,9(12,6-14,7)	0,396	0,204	0,370	0,435
Microalbuminúria, mg/L (mediana)	11,2(5,9-23,4)	29,5(10,1-58,7)	11,1(3,9-31,1)	14,3(6,6-38,3)	0,265	0,178	0,457	0,371
TFGe, mL/min/1,73m^3^ (mediana)	83,5(71,6-96,1)	76,3(47-103,1)	84,1(52,7-100,7)	69,4(50,5-89,1)	0,009	0,888	0,303	0,427
RCA, mg/g (mediana)	9,7(5,6-22,4)	40,1(7,8-78,5)	19,8(5,9-33,3)	15,7(8,6-45,2)	0,051	0,270	0,821	0,385
Diabetes (%)	24	27	0	27	0,341	0,074	0,068	0,969
Hipertensão (%)	70	91	90	90	0,028	0,943	1,000	0,931
DAC (%)	7,5	27	10	27	0,001	0,314	0,274	0,969
DRC (%)	8,9	27,3	40	33,3	<0,0001	0,537	0,702	0,712
IECA/BRA (%)	38	64	70	47	0,184	0,757	0,411	0,565
Betabloqueadores (%)	14	36	30	30	0,012	0,757	1,000	0,698
Diuréticos (%)	34	36	50	53	0,148	0,528	0,855	0,335
Desfecho composto, n (%)	39 (7,7)	7(63,6)	3(30)	15(50)	<0,0001	0,123	0,271	0,438

BNP: peptídeo natriurético tipo B; bpm: batimentos por minuto; BRA: bloqueador do receptor de angiotensina; DAC: doença arterial coronariana; DRC: doença renal crônica; FC: frequência cardíaca; IC: insuficiência cardíaca; ICFEi: insuficiência cardíaca com fração de ejeção intermediária; ICFEp: insuficiência cardíaca com fração de ejeção preservada; ICFEr: insuficiência cardíaca com fração de ejeção reduzida; IECA: inibidor da enzima de conversão da angiotensina; IMC: índice de massa corporal; PA: pressão arterial; RCA: relação creatinina-albumina; TFGe: taxa de filtração glomerular estimada. As variáveis categóricas são apresentadas como porcentagem (%) e as variáveis contínuas como mediana e intervalo interquartil (25% e 75%); o valor p geral para variáveis contínuas foi calculado com o teste de Kruskal-Wallis; as diferenças entre ICFEp, ICFEi e ICFEr foram calculadas usando o teste de Mann-Whitney; os valores de p para variáveis categóricas foram calculados usando o qui-quadrado de Pearson.

Ao analisar os parâmetros ecocardiográficos, a razão E/e’ média, o IMVE, o IVAE e o IVDF apresentaram diferença estatística na análise geral, com p < 0,001 em todas as análises. Os IMVE, IVAE e IVDF apresentaram valores intermediários no grupo ICFEi. O IMVE no grupo ICFEi foi menor do que no grupo ICFEr e semelhante ao grupo ICFEp. O IVAE no grupo ICFEi foi significativamente menor do que no grupo ICFEr e semelhante ao grupo ICFEp. O IVDF foi mais alto no grupo ICFEi quando comparado ao grupo ICFEp e menor quando comparado ao grupo ICFEr. Além disso, quando a razão E/e’ média em ICFEi e ICFEr foram analisados separadamente, a razão E/e’ do grupo ICFEi foi menor do que a do grupo ICFEr. (Tabela 2)

**Tabela 2:** Características clínicas de pacientes com insuficiência cardíaca, de acordo com fenótipo ICFEp, ICFEi ou ICFEp

	Sem IC (n=509)	ICFEr (n=11)	ICFEi (n=10)	ICFEp (n=30)	Geral	ICFEr vs. ICFEi	ICFEp vs. ICFEi	ICFEr vs. ICFEp
Fração de ejeção, %	61(58-65)	29(23-33)	43,5(41-48)	59,5(56,7-64,2)	<0,0001	<0,0001	<0,0001	<0,0001
Razão E/e' média, (±SD)	6,5(5,4-7,8)	9,6(7,5-17)	8,3(6-9,1)	7,9(6,1-12,1)	<0,0001	0,149	0,791	0,162
IVAE, ml/m^2^, (±SD)	20,9(17,3-24,5)	38,6(26,8-65,9)	30,5(18,9-42,2)	29,4(24,3-41,8)	<0,0001	0,231	0,607	0,188
IMVE, g/m^2^, (±SD)	89,3(76,5-102,8)	160,2(113,1-187,3)	119,0(102,9-154,0)	104,2(76,9-127,1)	<0,0001	0,091	0,123	0,002
IVDF, ml/m^2^, (±SD)	62,8(54,5-71,2)	106,0(82,5-150,3)	93,8(75,6-114,3)	68,7(54,2-76,2)	<0,0001	0,360	<0,0001	0,001

E: velocidade de fluxo mitral precoce; E´: velocidade diastólico precoce do anel mitral; IC: insuficiência cardíaca; ICFEi: insuficiência cardíaca com fração de ejeção intermediária; ICFEp: insuficiência cardíaca com fração de ejeção preservada; ICFEr: insuficiência cardíaca com fração de ejeção reduzida; IVAE: índice de volume do átrio esquerdo; IVDF: índice de volume diastólico final; IMVE: índice de massa do ventrículo esquerdo. Os dados são apresentados como mediana e intervalo interquartil (25% e 75%); (*) o valor p geral foi calculado pelo teste de Kruskal-Wallis; as diferenças entre ICFEp, ICFEi e ICFEr foram calculadas usando o teste de Mann-Whitney.

### Prognóstico de fenótipos de IC

**Figura 2:**
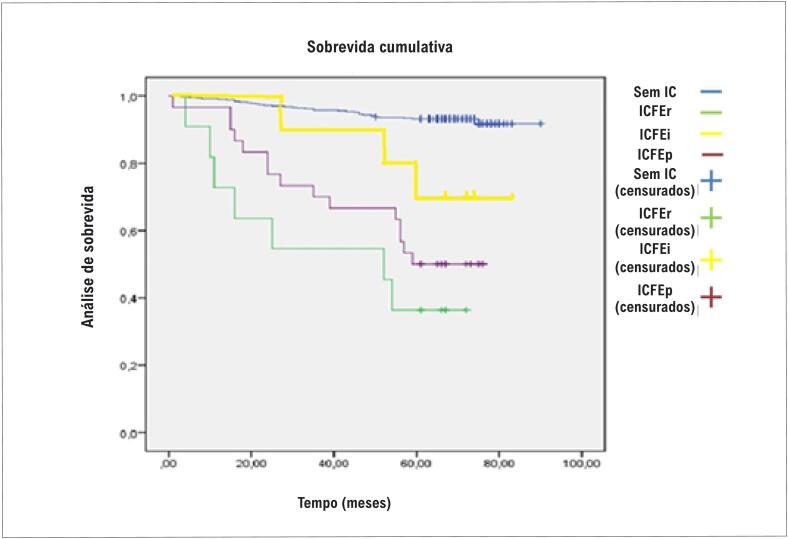
Curvas de Kaplan-Meier mostram que pacientes com ICFEi tiveram um pior desfecho composto de óbito por todas as causas e internação cardiovascular do que pacientes sem IC (p < 0,007), mas pacientes com ICFEi tiveram melhor prognóstico em comparação com pacientes com ICFEp e ICFEr (p < 0,001). O ICFEr teve o pior prognóstico dos três fenótipos de IC. IC: insuficiência cardíaca; ICFEi: IC com fração de ejeção intermediária; ICFEp: IC com fração de ejeção preservada; ICFEr: IC com fração de ejeção reduzida.

Após 5 anos, ocorreram 64 desfechos compostos, especificamente, 50 óbitos e 14 internações hospitalares por doença cardiovascular. Na curva de Kaplan-Meier (Figura 2), os pacientes com ICFEi apresentaram pior desfecho composto de óbito por todas as causas e interação cardiovascular do que os pacientes sem IC. No entanto, os pacientes com ICFEi tiveram melhor prognóstico na análise de Kaplan-Meier, quando comparados aos pacientes com ICFEp e ICFEr, enquanto os pacientes com ICFEp tiveram melhor prognóstico do que aqueles com fenótipo ICFEr. A Tabela 3 mostra as médias e seus intervalos de confiança de sobrevivência para os diferentes fenótipos de IC.

**Tabela 3:** Média e intervalo de confiança das probabilidades de sobrevivência em fenótipos de insuficiência cardíaca

		Intervalo de confiança 95%	
Variáveis	Estimativa média	Limite inferior	Limite superior
Sem IC	85,74	84,357	87,134
ICFEr	41,81	25,646	57,990
ICFEi	72,00	60,544	83,456
ICFEp	54,56	45,561	63,572

IC: insuficiência cardíaca; ICFEi: IC com fração de ejeção intermediária; ICFEp: IC com fração de ejeção preservada; ICFEr: IC com fração de ejeção reduzida.

## Discussão

Desde a adoção de ICFEi como um novo fenótipo de IC, o desafio maior tem sido definir as características de linha de base, a fisiopatologia e o tratamento para este novo grupo de pacientes. O presente artigo é o primeiro estudo do fenótipo de ICFEi em uma população brasileira, envolvendo pacientes da atenção primária. Realizamos uma análise do estudo Digitalis^10^ com a finalidade de avaliar a prevalência e as características clínicas e ecocardiográficas de pacientes com ICFEi no Brasil.

Em nossa população de pacientes com IC, a prevalência de ICFEi foi de 22%, semelhante a outros estudos.^6^^-^^8^

Os estudos de Rickenbacher et al.^12^ e Tsuji et al.,^7^ mostraram que os níveis de BNP eram intermediários na ICFEi. Porém, em nosso estudo, o BNP no grupo ICFEi não apresentou valores intermediários; foi semelhante ao grupo ICFEp e apresentou valores inferiores aos do grupo ICFEr. Porém, em relação à prevalência de etiologia isquêmica no grupo ICFEi, nosso estudo mostrou que ICFEi foi semelhante a ICFEr, semelhante a estudos anteriores. Resultados do estudo de Kapoor et al.^6^ e o registro sueco de IC^11^ sugerem que a etiologia isquêmica é distintamente mais comum na ICFEr e na ICFEi. O estudo TOPCAT^13^ avaliou o uso da espironolactona em pacientes com diferentes faixas de FEVE e mostrou que houve redução de internações em pacientes com IC, principalmente aqueles com FEVE entre 45% e 50%. No estudo CHARM^14^ concluiu que o uso de candesartana melhorou os desfechos, tanto para ICFEi quanto para ICFEr. Assim, por extrapolação, ICFEi poderia responder ao tratamento recomendado para ICFEr de etiologia isquêmica, conforme sugerido pelas diretrizes do IC.^3^^,^^5^

Ao analisar os parâmetros da Doppler ecocardiografia, o IMVE, o IVAE e a razão E/e’, no grupo ICFEi, foram semelhantes ao grupo ICFEp, enquanto o IVDF no grupo ICFEi apresentou valores intermediários, com diferenças estatísticas quando comparado aos grupos ICFEp e ICFEr. O estudo de Rastogi et al.,^15^ sugere que os pacientes com ICFEi são um grupo heterogêneo, com pelo menos 3 subgrupos com base na FEVE, a saber, pacientes com FEVE prévia < 40% (fração de ejeção recuperada), pacientes com FEVE prévia > 50% (fração de ejeção deteriorada) e pacientes com FEVE prévia entre 40% e 50% (fração de ejeção inalterada). Esses achados reforçam a ideia de que a fisiopatologia da ICFEi pode ter uma contribuição da disfunção sistólica e uma contribuição da disfunção diastólica, conforme sugerido pelas diretrizes da Sociedade Europeia de Cardiologia de 2016.^4^

Em relação ao prognóstico, nosso estudo concluiu que os pacientes com ICFEi apresentaram melhor desfecho composto de mortalidade por todas as causas e internação cardiovascular melhor do que aqueles com ICFEr e ICFEp (p < 0,001). Nossos resultados estão de acordo com uma meta-análise de Altaie et al.^9^ que mostrou que o fenótipo ICFEi teve uma taxa de mortalidade por todas as causas significativamente menor do que ICFEr (RR, 0,9; intervalo de confiança de 95%, 0,85 a 0,94; p < 0,001). No entanto, diferentemente do presente estudo, não verificaram nenhuma diferença significativa na mortalidade por todas as causas entre ICFEp e ICFEi (RR, 0,98; intervalo de confiança de 95%, 0,86 a 1,12; p = 0,82).^9^ Na meta-análise por Altaie et al., analisando internação devido à IC, não encontraram diferenças significativas entre ICFEr e ICFEi (RR, 0,92; intervalo de confiança de 95%, 0,84 a 1,01; p = 0,08) ou entre ICFEp e ICFEi (RR, 1,05; intervalo de confiança de 95%, 0,83 a 1,33; p = 0,69).

São necessários futuros estudos que investiguem o prognóstico e caracterizem a ICFEi com uma amostra maior. Além disso, o presente estudo abre caminho para futuros ensaios clínicos randomizados que investiguem tratamentos específicos para pacientes com ICFEi.

### Limitações

Os resultados devem ser interpretados com várias limitações. Primeiramente, foi avaliado um pequeno número de pacientes com IC, o que pode não representar toda a população. Em segundo lugar, a avaliação clínica e as variáveis laboratoriais e ecocardiográficas, incluindo a FEVE, foram baseadas em uma única medida. Além disso, embora as características sociodemográficas da população do estudo sejam bastante semelhantes às de outras áreas urbanas em todo o mundo, as extrapolações destes resultados devem ser feitas com cautela. Por fim, como a população do estudo era composta por voluntários, é possível que algum viés de seleção tenha sido introduzido, por exemplo, maior percentual de mulheres.

## Conclusão

A prevalência de ICFEI foi semelhante ao observado em estudos anteriores. O presente estudo demonstrou que a ICFEi apresenta características clínicas e ecocardiográficas mais semelhantes à ICFEp, do que à ICFEr. Além disso, nossos dados mostram que a ICFEi teve melhor prognóstico em comparação com os outros dois fenótipos.
