# Supplementary material for: Características e Tendências na Mortalidade em Diferentes Fenótipos de Insuficiência Cardíaca na Atenção Primária
Source: Arq Bras Cardiol. 2021 Aug 9;117(2):300–6. [Article in Portuguese] doi: 10.36660/abc.20190912 (PMC8395788; doi:10.36660/abc.20190912)
Supplement: Translation

# Translation: Characteristics and Temporal Trends in the Mortality of Different Heart Failure Phenotypes in Primary Care

**Authors:** Antonio José Lagoeiro Jorge, Leticia Mara dos Santos Barbetta, Eduardo Thadeu de Oliveira Correia, Wolney de Andrade Martins, Adson Renato Leite, Maria Auxiliadora Nogueira Saad, Márcia Maria Sales dos Santos, Dayse Mary Correia, Maria Luiza Garcia Rosa, Sérgio Chermont, Cárita Cunha dos Santos, Evandro Tinoco Mesquita

**Affiliations:** 1 NiteróiRJ Brazil Universidade Federal Fluminense, Niterói, RJ - Brazil.; 2 Brazil C.T.E.B, UHG, - Brazil.

**Keywords:** Heart Failure/trends, Heart Failure/mortality, Prevalence, Primary Health Care, Prognosis, Eidemiology, Stroke Volume

## Abstract

**Background:**

The classification of heart failure (HF) by phenotypes has a great relevance in clinical practice.

**Objective:**

The study aimed to analyze the prevalence, clinical characteristics, and outcomes between HF phenotypes in the primary care setting.

**Methods:**

This is an analysis of a cohort study including 560 individuals, aged ≥ 45 years, who were randomly selected in a primary care program. All participants underwent clinical evaluations, b-type natriuretic peptide (BNP) measurements, electrocardiogram, and echocardiography in a single day. HF with left ventricular ejection fraction (LVEF) < 40% was classified as HF with reduced ejection fraction (HFrEF), LVEF 40% to 49% as HF with mid-range ejection fraction (HFmrEF) and LVEF ≥ 50% as HF with preserved ejection fraction (HFpEF). After 5 years, the patients were reassessed as to the occurrence of the composite outcome of death from any cause or hospitalization for cardiovascular disease.

**Results:**

Of the 560 patients included, 51 patients had HF (9.1%), 11 of whom had HFrEF (21.6%), 10 had HFmrEF (19.6%) and 30 had HFpEF (58.8%). HFmrEF was similar to HFpEF in BNP levels (p < 0.001), left ventricular mass index (p = 0.037), and left atrial volume index (p < 0.001). The HFmrEF phenotype was similar to HFrEF regarding coronary artery disease (p = 0.009). After 5 years, patients with HFmrEF had a better prognosis when compared to patients with HFpEF and HFrEF (p < 0.001).

**Conclusion:**

The prevalence of ICFEI was similar to that observed in previous studies. ICFEI presented characteristics similar to ICFEP in this study. Our data show that ICFEi had a better prognosis compared to the other two phenotypes.

## Introduction

The classification of heart failure (HF) by phenotypes has great relevance in clinical practice, since they differ in relation to the characteristics, prognosis, and treatment of the patient.^1^ Classically, two phenotypes of HF were described in guidelines, namely, HF with reduced ejection fraction (HFrEF) where left ventricular ejection fraction (LVEF) is less than 50% and HF with preserved ejection fraction (HFpEF) with LVEF ≥ 50%.^2^ In 2013, the American College of Cardiology Foundation/American Heart Association published new guidelines for HF, in which patients with LVEF between 41% and 50% were classified as borderline HFpEF.^3^ In 2016, the HF guidelines of the European Society of Cardiology recognized HF with LVEF between 40% and 49% as a distinct phenotype, called HF with mid-range ejection fraction (HFmrEF).^4^ Finally, in 2018, the Brazilian Society of Cardiology added HFmrEF to the 2018 Chronic and Acute Heart Failure Guidelines.^5^

Recent studies have observed that the prevalence of patients with HFmrEF ranged from 13% to 24% of all patients with HF.^6^^-^^8^ Current data from HF studies indicates that HFmrEF presents intermediate characteristics.^8^ Moreover, a meta-analysis that included more than 600,000 patients with HF concluded that patients with HFmrEF had lower all-cause mortality than HFrEF patients and no statistical difference from patients with HFpEF. Regarding all-cause hospitalization, there was no statistical difference between all the three HF phenotypes.^9^ There are no studies in Brazil that have evaluated this phenotype in primary care. Therefore, the present study aimed to analyze the prevalence and the clinical characteristics of HFmrEF, as well as the outcomes among HF phenotypes in patients from a primary care setting.

## Methods

This cohort study included, at the baseline, 633 individuals aged ≥ 45 years, who were registered in the Primary Care Program of the city of Niterói, a medium-sized city with 487,562 inhabitants in the state of Rio de Janeiro, Brazil. The Primary Care Program offers coverage to 137,463 residents in 32 service modules, divided into 110 sectors. Initially, 21 sectors were selected through a random sequence, generated by a computer program, in which the weight of each sector was proportional to the number of individuals.^10^ The data were collected from July 2011 to December 2012. After 5 years, the patients in this study were reassessed as to the occurrence of the composite outcome of death from any cause or hospitalization for cardiovascular disease. During the follow-up, there were 73 (11.5%) losses, and the final number of individuals assessed was 560.

### Population

The survey sample size was estimated based on a minimum HF prevalence of 6%, with an absolute error of 2% (confidence interval [CI] = 99%, 4% to 8%). This assumption required a sample size of 580 individuals. In each one of the 21 sectors included, 30 individuals between 45 and 100 years of age were randomly chosen. Another 20 individuals per unit were also chosen to allow replacement in case of impossibility of participation, totaling 1,050 selected individuals. In this manner, we sent letters to health unit staff to invite 1,050 individuals to participate in this study, and 666 of these individuals attended the visit and signed the consent form. Thirty-three individuals who did not complete all of the research procedures were excluded. The baseline population was 633 individuals, 73 (11.5%) of whom were not located after 5 years and were subsequently excluded. The final population was 560 individuals. (Figure 1)

**Figure 1:** Population selection flowchart.

The choice of the primary care units and the number of individuals in each unit were planned in order to represent the demographic distribution. The selection of subjects was carried out through a random sequence generated by a computer program. The inclusion criteria were age ≥ 45 years and willingness to provide informed consent. Whenever there was a refusal, the next subject in the randomized list was invited to participate.

All participants in the study underwent a single-day evaluation that consisted of the following: (a) anamnesis and clinical examinations; (b) laboratory tests, including b-type natriuretic peptide (BNP) dosage; (c) 12-lead electrocardiogram (ECG); and (d) tissue Doppler echocardiography. ECG was performed in 12 simultaneous leads. Tissue Doppler echocardiography was performed by two certified physicians, using two portable devices, the Acuson Cypress 20 (Siemens, USA) and the AU-3 Partner (Esaote, Italy). The physicians were blinded from the clinical status and exam results. The exams were performed according to the recommendations of the quantification of chambers from the American Society of Echocardiography and the European Association of Echocardiography.^11^ Systolic function was assessed by measuring LVEF using Simpson's method.

### Definition of heart failure phenotypes

Diagnosis of HFrEF was confirmed in individuals with a history of HF or the presence of signs or symptoms of HF and LVEF < 40%. Diagnosis of HFpEF was confirmed in individuals with a history of HF or signs or symptoms of HF with LVEF ≥ 50% and end-diastolic volume index (EDVI) < 97 mL/m^2^, in the presence of diastolic dysfunction of the left ventricle and BNP > 35 pg/mL. HFmrEF was confirmed in individuals with a history of HF or signs or symptoms of HF with LVEF between 40% and 49% and BNP > 35 pg/mL.^4^^,^^12^

### Statistical analysis

Continuous variables were expressed as median and interquartile range, as none of them were positive for normality when tested using the Kolmogorov-Smirnov test. Categorical variables were summarized as absolute and relative frequencies. Regarding quantitative variables, differences between HF phenotypes were tested with the non-parametric tests (Mann-Whitney and Kruskal-Wallis), while categorical variables were assessed by chi-squared test. A Kaplan-Meier curve was estimated for the composite outcomes of the four possibilities (HF-free, HFrEF, HFmrEF, and HFpEF). The difference between the four curves and between HFmrEF and HF-free were tested with the log rank test. P values < 0.05 were considered statistically significant. All statistical analysis was performed with SPSS software version 23.0 (Chicago, Illinois, USA).

### Ethical considerations

This study was conducted in accordance with the principles of the Declaration of Helsinki revised in 2000. The study protocol was approved by the institution's Ethics Committee under number 0077.0.258.000-10.

## Results

### Prevalence and characteristics of patients with HFmrEF

Of the 560 patients included in the study, 509 were not diagnosed with HF (90.9%), and 51 were diagnosed with HF (9.1%). Of the 51 patients with HF, 11 had HFrEF (21.6%), 10 had HFmrEF (19.6%), and 30 had HFpEF (58.8%). The demographic and clinical characteristics of patients with HF are shown in Table 1. HFmrEF was similar to HFpEF in left ventricular mass index (LVMI) and left atrial volume index (LAVI). We observed more coronary artery disease in patients with the HFrEF phenotype, compared to HFmrEF. The percentage of chronic kidney disease was intermediate in the HFmrEF group, being lower than HFpEF and higher than HFrEF. The HFmrEF group had intermediate values in following characteristics: heart rate, glucose levels, and creatinine-albumin ratio. However, there was no statistical difference among the groups with HF in these characteristics.

**Table 1:** Demographic and clinical characteristics of patients with heart failure, according to phenotype HFpEF, HFmrEF, or HFpEF

	HF-free (n=509)	HFrEF (n=11)	HFmrEF (n=10)	HFpEF (n=30)	Overall	HFrEF vs. HFmrEF	HFpEF vs. HFmrEF	HFrEF vs. HFpEF
Male sex (%)	37	64	40	27	0.190	0.279	0.426	0.029
Age, years (median)	57(51-64)	74(57-78)	72(60-79)	72.5(64.7-81.7)	<0.001	0.809	0.708	0.871
BMI (median)	27.2(24.5-30.8)	24.9(21.3-25.9)	28.1(26.3-30.6)	26.9(22.0-30.7)	0.156	0.057	0.319	0.496
HR, bpm (median)	70.5(63.2-77.5)	69(55.5-72.5)	72 (62.1-79.1)	76.5(63.2-84.7)	0.360	0.324	0,573	0.108
Systolic BP, mmHg (median)	133.3(121-147.5)	146(116-161)	130(117.9-157.8)	151.7(135.2-179.7)	0.001	0.751	0.032	0.168
Diastolic BP, mmHg (median)	82(74.1-90)	80(68.3-88.5)	77.5(71.1-90.9)	83.7(72.7-91.3)	0.699	0.778	0.699	0.310
BNP, pg/mL (median)	15(10-25)	306(153-615)	61.5(51-95)	87.5(52.7-120.5)	<0.0001	0.002	0.281	0.001
Glucose, mg/dL (median)	100(91-113)	103(84-119)	97(87-106.2)	100(94.7-119)	0.765	0.621	0.288	0.757
Uric acid, mg/dL (median)	5.1(4.2-6.1)	6.3(4.6-8.0)	5.2(4.9-6.5)	5.1(4.1-6.7)	0.192	0.398	0.430	0.108
Total cholesterol, mg/dL (median)	213(186-244)	185(177-253)	199(180-240)	208(196-231)	0.629	0.623	0.453	0.502
Triglycerides, mg/dL (median)	118(86-169)	115(86-190)	106(66-152)	101(90-136)	0.481	0.571	0.851	0.482
Hemoglobin, g/dL (median)	13.7(12.8-14.7)	13.9(13.4-16.4)	13.7(12.1-14.3)	13.9(12.6-14.7)	0.396	0.204	0.370	0.435
Microalbuminuria, mg/L (median)	11.2(5.9-23.4)	29.5(10.1-58.7)	11.1(3.9-31.1)	14.3(6.6-38.3)	0.265	0.178	0.457	0.371
eGFR, mL/min/1.73m^3^ (median)	83.5(71.6-96.1)	76.3(47-103.1)	84.1(52.7-100.7)	69.4(50.5-89.1)	0.009	0.888	0.303	0.427
CAR, mg/g (median)	9.7(5.6-22.4)	40.1(7.8-78.5)	19.8(5.9-33.3)	15.7(8.6-45.2)	0.051	0.270	0.821	0.385
Diabetes (%)	24	27	0	27	0.341	0.074	0.068	0.969
Hypertension (%)	70	91	90	90	0.028	0.943	1.000	0.931
CAD (%)	7.5	27	10	27	0.001	0.314	0.274	0.969
CKD (%)	8.9	27.3	40	33.3	<0.0001	0.537	0.702	0.712
ACEI/ARB (%)	38	64	70	47	0.184	0.757	0.411	0.565
Beta-blockers (%)	14	36	30	30	0.012	0.757	1.000	0.698
Diuretics (%)	34	36	50	53	0.148	0.528	0.855	0.335
Composite outcome, n (%)	39 (7.7)	7(63.6)	3(30)	15(50)	<0.0001	0.123	0.271	0.438

ACEI: angiotensin-converting enzyme inhibitor; ARB: angiotensin receptor blocker; BMI: body mass index; BNP: type B natriuretic peptide; BP: blood pressure; bpm: beats per minute; CAD: coronary artery disease; CAR: creatinine-albumin ratio; CKD: chronic kidney disease; eGFR: estimated glomerular filtration rate; HF: heart failure; HFmrEF: heart failure with mid-range ejection fraction; HFpEF: heart failure with preserved ejection fraction; HFrEF: heart failure with reduced ejection fraction; HR: heart rate. Categorical variables are shown as percentage (%) and continuous variables as median and interquartile range (25% and 75%); overall p value for continuous variables were calculated using the Kruskal-Wallis test; differences between HFpEF, HFmrEF, and HFrEF were calculated using the Mann-Whitney test; p values for categorical variables were calculated using Pearson's chi-square.

When analyzing the echocardiography parameters, the mean E/e’ ratio, LVMI, LAVI, and EDVI showed statistical difference in overall analysis, with p < 0.001 in all analyses. The LVMI, LAVI, and EDVI showed intermediate values in HFmrEF. The LVMI in HFmrEF was lower than in HFrEF and similar to HFpEF. The LAVI in HFmrEF was significantly lower than HFrEF and similar to HFpEF. The EDVI was higher in HFmrEF when compared to HFpEF and lower when compared to HFrEF. Moreover, when the mean E/e’ ratio in HFmrEF and HFrEF were analyzed separately, the E/e’ ratio of the HFmrEF group was lower than that of the HFrEF group. (Table 2)

**Table 2:** Clinical characteristics of patients with heart failure, according to phenotype HFpEF, HFmrEF, or HFpEF

	HF-free (n=509)	HFrEF (n=11)	HFmrEF (n=10)	HFpEF (n=30)	Overall	HFrEF vs. HFmrEF	HFpEF vs. HFmrEF	HFrEF vs. HFpEF
Ejection fraction, %	61(58-65)	29(23-33)	43.5(41-48)	59.5(56.7-64.2)	<0.0001	<0.0001	<0.0001	<0.0001
Mean E/e' ratio, (±SD)	6.5(5.4-7.8)	9.6(7.5-17)	8.3(6-9.1)	7.9(6.1-12.1)	<0.0001	0.149	0.791	0.162
LAVI, ml/m^2^, (±SD)	20.9(17.3-24.5)	38.6(26.8-65.9)	30.5(18.9-42.2)	29.4(24.3-41.8)	<0.0001	0.231	0.607	0.188
LVMI, g/m^2^, (±SD)	89.3(76.5-102.8)	160.2(113.1-187.3)	119.0(102.9-154.0)	104.2(76.9-127.1)	<0.0001	0.091	0.123	0.002
EDVI, ml/m^2^, (±SD)	62.8(54.5-71.2)	106.0(82.5-150.3)	93.8(75.6-114.3)	68.7(54.2-76.2)	<0.0001	0.360	<0.0001	0.001

E: early mitral inflow velocity; E´: mitral annular early diastolic velocity; EDVI: end-diastolic volume index; HF: heart failure; HFmrEF: heart failure with mid-range ejection fraction; HFpEF: heart failure with preserved ejection fraction; HFrEF: heart failure with reduced ejection fraction; LAVI: left atrial volume index; LVMI: left ventricular mass index. Data are shown as median and interquartile range (25% and 75%); (*) overall p value were calculated using the Kruskal-Wallis test; differences between HFpEF, HFmrEF and HFrEF were calculated using the Mann-Whitney test.

### Prognosis of HF phenotypes

After 5 years, 64 composite outcomes occurred, namely, 50 deaths and 14 hospitalizations for cardiovascular disease. In the Kaplan-Meier curve (Figure 2), patients with HFmrEF had a worse composite outcome of all-cause death and cardiovascular hospitalization than patients without HF. However, patients with HFmrEF had a better prognosis in the Kaplan-Meier analysis, when compared to patients with HFpEF and HFrEF, whereas patients with HFpEF had better prognosis than those with the HFrEF phenotype. Table 3 shows the means and their confidence intervals of survival for the different HF phenotypes.

**Figure 2:** Kaplan-Meier curve showing that patients with HFmrEF had a worse composite outcome of all-cause death and cardiovascular hospitalization than patients without HF (p < 0.007), but patients with HFmrEF had better prognosis compared to patients with HFpEF and HFrEF (p < 0.001). HFrEF had the worst prognosis of the three phenotypes of HF. HF: heart failure; HFmrEF: HF with mid-range ejection fraction; HFpEF: HF with preserved ejection fraction; HFrEF: HF with reduced ejection fraction.

**Table 3:** Mean and confidence interval of survival probabilities in heart failure phenotypes

		Confidence interval 95%	
Variables	Means estimate	Lower limit	Upper limit
No HF	85.74	84.357	87.134
HFrEF	41.81	25.646	57.990
HFmrEF	72.00	60.544	83.456
HFpEF	54.56	45.561	63.572

HF: heart failure; HFmrEF: HF with mid-range ejection fraction; HFpEF: HF with preserved ejection fraction; HFrEF: HF with reduced ejection fraction.

## Discussion

Since the adoption of HFmrEF as a new phenotype of HF, the major challenge has been to define the baseline characteristics, pathophysiology, and treatment for this new group of patients. The present article is the first study of HFmrEF in a Brazilian population, involving primary care patients. We conducted an analysis of the Digitalis study^10^ in order to evaluate the prevalence and the clinical and echocardiographic characteristics of patients with HFmrEF in Brazil.

In our population of patients with HF, the prevalence of HFmrEF was 22%, similar to other studies.^6^^-^^8^

The studies by Rickenbacher et al.^12^ and Tsuji et al.^7^ showed that BNP levels were intermediate in HFmrEF. However, in our study, BNP in the HFmrEF group did not present intermediate values; it was similar to HFpEF, and it showed lower values than in HFrEF. However, regarding the prevalence of ischemic etiology in the HFmrEF group, our study showed that HFmrEF was similar to HFrEF, similar to previous studies. Results from the study by Kapoor et al.^6^ and the Swedish-HF registry^11^ suggest that ischemic etiology is distinctly more common in HFrEF and HFmrEF. The TOPCAT study^13^ evaluated the use of spironolactone in patients with different LVEF ranges and showed that there was a reduction in hospitalizations in patients with HF, especially those with LVEF between 45% and 50%. In the CHARM study,^14^ it was concluded that the use of candesartan improved the outcomes for HFmrEF as well as HFrEF. Thus, by extrapolation, HFmrEF could respond to the recommended treatment for HFrEF of ischemic etiology, as suggested by the HF guidelines.^3^^,^^5^

When analyzing the parameters of Doppler echocardiography, the LVMI, LAVI, and the E/e’ ratio, in the HFmrEF group, were similar to HFpEF, while the EDVI in HFmrEF showed intermediate values, with statistical differences when compared to HFpEF and HFrEF. The study by Rastogi et al.^15^ suggests that patients with HFmrEF are a heterogeneous group, with at least 3 subgroups based on LVEF, namely, patients with previous LVEF < 40% (recovered ejection fraction), patients with previous LVEF > 50% (deteriorated ejection fraction), and patients with previous LVEF between 40% and 50% (unchanged ejection fraction). These findings reinforce the idea that the physiopathology of HFmrEF may have a contribution of systolic dysfunction and a contribution of diastolic dysfunction, as suggested by the 2016 European Society of Cardiology guidelines.^4^

Regarding prognosis, our study concluded that patients with HFmrEF had a better composite outcome of all-cause mortality and cardiovascular hospitalization than those with HFrEF and HFpEF (p < 0.001). Our results are in agreement with a meta-analysis by Altaie et al.^9^ that showed that the HFmrEF phenotype had a significantly lower all-cause death rate than the HFrEF (RR, 0.9; 95% CI, 0.85 to 0.94; p < 0.001). However, differently from the present study, they found no significant difference between the all-cause mortality of HFpEF and HFmrEF (RR, 0.98; 95% CI, 0.86 to 1.12; p = 0.82).^9^ Analyzing hospitalization due to HF in the meta-analysis by Altaie et al., they found no significant differences between HFrEF and HFmrEF (RR, 0.92; 95% CI, 0.84 to 1.01; p = 0.08) or between HFpEF and HFmrEF (RR, 1.05; 95% CI, 0.83 to 1.33; p = 0.69).

Further studies that investigate the prognosis and characterize HFmrEF with a larger sample are necessary. In addition, the present study paves the way for future randomized trials that investigate specific treatments for patients with HFmrEF.

### Limitations

The results should be interpreted with several limitations. First, a small number of patients with HF were evaluated, which may not represent the whole population. Second, clinical evaluation and laboratory and echocardiographic variables, including LVEF, were based on a single measurement. Furthermore, although the sociodemographic characteristics of the studied population are quite similar to other urban areas worldwide, extrapolations of these results should be taken with caution. Lastly, since the study population comprised volunteers, it is possible that some selection bias was introduced, such as higher percentage of women.

## Conclusion

The prevalence of ICFEI was similar to that observed in previous studies. The present study demonstrated that ICFEi has clinical and echocardiographic characteristics that are more similar to ICFEp than to ICFEr. In addition, our data show that ICFEi had a better prognosis compared to the other two phenotypes.
